# Mitochondrial Gene Expression Is Responsive to Starvation Stress and Developmental Transition in *Trypanosoma cruzi*

**DOI:** 10.1128/mSphere.00051-16

**Published:** 2016-04-13

**Authors:** Aubie K. Shaw, Murat C. Kalem, Sara L. Zimmer

**Affiliations:** Department of Biomedical Sciences, University of Minnesota Medical School, Duluth, Minnesota, USA; University of Georgia

**Keywords:** *Kinetoplastida*, RNA editing, *Trypanosoma cruzi*, mRNA stability, mitochondria, regulation of gene expression

## Abstract

*Trypanosoma cruzi* parasites causing Chagas disease are passed between mammals by the triatomine bug vector. Within the insect, *T. cruzi* epimastigote-stage cells replicate and progress through the increasingly nutrient-restricted digestive tract, differentiating into infectious, nonreplicative metacyclic trypomastigotes. Thus, we evaluated how nutrient perturbations or metacyclogenesis affects mitochondrial gene expression in different insect life cycle stages. We compared mitochondrial RNA abundances in cultures containing fed, replicating epimastigotes, differentiating cultures containing both starved epimastigotes and metacyclic trypomastigotes and epimastigote starvation cultures. We observed increases in mitochondrial rRNAs and some mRNAs in differentiating cultures. These increases predominated only for the edited CYb mRNA in cultures enriched for metacyclic trypomastigotes. For the other transcripts, abundance increases were linked to starvation and were strongest in culture fractions with a high population of starved epimastigotes. We show that loss of both glucose and amino acids results in rapid increases in RNA abundances that are quickly reduced when these nutrients are returned. Furthermore, the individual RNAs exhibit distinct temporal abundance patterns, suggestive of multiple mechanisms regulating individual transcript abundance. Finally, increases in mitochondrial respiratory complex subunit mRNA abundances were not matched by increases in abundances of nucleus-encoded subunit mRNAs, nor were there statistically significant increases in protein levels of three nucleus-encoded subunits tested. These results show that, similarly to that in *T. brucei*, the mitochondrial genome in *T. cruzi* has the potential to alter gene expression in response to environmental or developmental stimuli but for an as-yet-unknown purpose.

**IMPORTANCE** Chagas disease is caused by insect-transmitted *Trypanosoma cruzi*. Halting *T. cruzi*’s life cycle in one of its various human and insect life stages would effectively stop the parasite’s infection cycle. *T. cruzi* is exposed to a variety of environmental conditions in its different life stages, and gene expression must be remodeled to survive these changes. In this work, we look at the impact that one of these changes, nutrient depletion, has on the expression of the 20 gene products encoded in the mitochondrial genome that is neglected by whole-genome studies. We show increases in mitochondrial RNA abundances in starved insect-stage cells, under two conditions in which transition to the infectious stage occurs or does not. This report is the first to show that *T. cruzi* mitochondrial gene expression is sensitive to environmental perturbations, consistent with mitochondrial gene expression regulatory pathways being potential antiparasitic targets.

## Abstract (precis)

Chagas disease is caused by insect-transmitted *Trypanosoma cruzi*. Halting *T. cruzi*’s life cycle in one of its various human and insect life stages would effectively stop the parasite’s infection cycle. *T. cruzi* is exposed to a variety of environmental conditions in its different life stages, and gene expression must be remodeled to survive these changes. In this work, we look at the impact that one of these changes, nutrient depletion, has on the expression of the 20 gene products encoded in the mitochondrial genome that is neglected by whole-genome studies. We show increases in mitochondrial RNA abundances in starved insect-stage cells, under two conditions in which transition to the infectious stage occurs or does not. This report is the first to show that *T. cruzi* mitochondrial gene expression is sensitive to environmental perturbations, consistent with mitochondrial gene expression regulatory pathways being potential antiparasitic targets.

## INTRODUCTION

The various environmental conditions that pathogenic trypanosomatids encounter during their respective life cycles represent challenges to their survival. The environmental and functional demands of their various life cycle stages, as they are transmitted from insect vectors to humans and livestock, require major developmental remodeling of gene expression. Importantly, environmental stresses are sometimes necessary to signal life-stage differentiations for successful progression through the life cycle (1). Therefore, life-stage changes, responses to environmental stresses, and regulation of gene expression are intertwined.

Myriad studies have addressed changes in trypanosome gene expression by examining mRNA abundance, protein abundance, and translational efficiency between life stages or upon exposure to environmental stimuli (2, 3). Investigations in *Trypanosoma cruzi* alone are continually refining our understanding of its gene expression (4–9). These studies have focused on changes in nuclear gene expression. Yet important developmental differences have also been observed in trypanosome mitochondrial gene expression. For example, many mature *Trypanosoma brucei* mitochondrial RNAs are regulated in a life-stage-dependent manner, with differential editing (see below) playing a key role (10–14).

Trypanosome mitochondrial gene expression has repeatedly been identified as a potential target for antiparasitics, as it has many unique aspects (15–17). The mitochondrial DNA of trypanosomatids is compacted into the disk-shaped kinetoplast within the single organelle. Two rRNAs and 18 mRNAs are encoded on circular molecules within the kinetoplast called “maxicircles” from which transcription is polycistronic. The major mechanisms regulating mitochondrial gene expression are posttranscriptional (18) and include RNA stability, processing, and translation (18–27). A prominent and unique feature of trypanosome mitochondrial RNA processing is uridine insertion/deletion RNA editing, which results in mature translatable mRNAs (28).

Most of the overall mechanisms and details of mitochondrial gene expression have been worked out in the *T. brucei* and *Leishmania tarentolae* model systems (18, 28–31). In contrast, the study of *T. cruzi* mitochondrial gene expression and its regulation is still in its infancy. This is problematic, as there are important differences among the life cycles of these trypanosomes. For instance, *T. brucei* lacks an intracellular stage, and metacyclic differentiation is not influenced by progression through the insect digestive tract as it is in *T. cruzi* (32). Additionally, *T. brucei* stage-specific mitochondrial gene regulation is thought necessary because the *T. brucei* life cycle includes both the electron transport chain (ETC)-utilizing insect stage and the bloodstream stage in which oxidative phosphorylation is disabled (29, 33). In contrast, evidence supports the maintenance of a functional ETC throughout the *T. cruzi* life cycle (5, 33–36). Even with an ETC that is continuously present, *T. cruzi* alters its rate of glycolysis, utilization of Krebs cycle enzymes, oxidative phosphorylation, and proteolysis to obtain energy from different substrates encountered in different life cycle stages or environmental stresses (34, 37). This regulation may require modulation of the abundances or activities of ETC complexes, which are essential for *T. cruzi* energy generation (15, 38). In the mitochondrial genome, 15 of the 20 gene products are likely or confirmed subunits of ETC complexes and are thus perhaps developmentally regulated. Therefore, mitochondrial gene regulation throughout the life cycle stages of *T. cruzi* requires systematic analysis.

We investigated insect-stage *T. cruzi* mitochondrial gene expression regulation in development- and stress-related contexts. During the *T. cruzi* life cycle, the insect ingests mammalian *T. cruzi* stages, which differentiate to the epimastigote form and replicate. As nutrients from the blood meal are depleted, epimastigotes adhere to the hindgut, elongate, and undergo metacyclogenesis to the infectious form (39–41) to complete their life cycle in the mammalian host again. Our model is a new culture system of gradual nutrient depletion in which epimastigotes differentiate into metacyclic trypomastigotes, with a mixed morphology present at most times. We also investigate mitochondrial gene expression in a system of rapid transition to complete starvation in which metacyclogenesis does not occur.

We find that certain maxicircle-encoded RNAs increase in abundance under starvation conditions that appear to promote metacyclogenesis, with the largest expression changes occurring in cells that have not committed to differentiation. An important exception is the increased abundance of mature CYb, which is greater in cell populations enriched for metacyclic trypomastigotes than in those enriched for epimastigotes. RNA stability and regulation of editing both apparently play roles in increasing RNA abundances. Furthermore, depletion of all nutrients is important for driving these rapid and reversible increases. Under these conditions, we found no evidence of corresponding mRNA abundance increases of nucleus-encoded ETC subunits, and changes in protein levels of three of these subunits were too subtle to accurately capture by immunoblot analysis.

## RESULTS

### Establishment of an insect-stage culture differentiation protocol.

**FIGURE 1:**
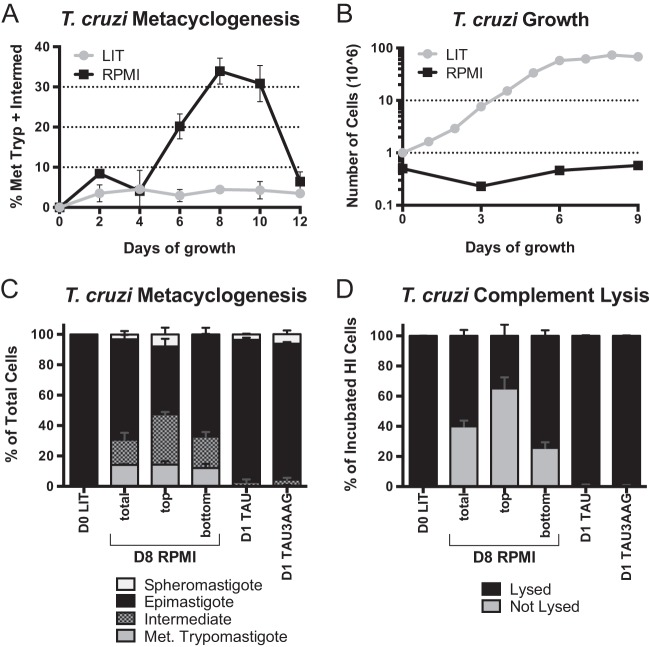
Characterization of cell stages and morphologies under the culture conditions used in this study. (A) DAPI-stained cells were examined with confocal microscopy. The percentages of intermediate (Intermed) plus metacyclic cells during 12 days in LIT or RPMI cultures are shown. Data points represent means and standard errors of the means (SEM) of results from 3 to 5 replicate flasks collected on 2 different days. Met Tryp, metacyclic trypomastigotes. (B) Nine-day growth curve of *T. cruzi* in LIT and RPMI cultures. Data points represent means and SEM of results from three replicate flasks; error bars are too small to be observed with this resolution. (C) DAPI-stained cells were examined with confocal microscopy. Data represent results of a stacked-column comparison of ratios of epimastigote, metacyclic trypomastigote, intermediate, and spheromastigote development in various media on the indicated days. Day 8 (D8) RPMI cultures (total) and the top and bottom fractions of a day 8 RPMI culture (top and bottom) were examined. Data points represent means and SEM of results from 3 to 11 replicate flasks collected on 3 different days. (D) Complement-mediated lysis assay. Stacked-column comparison of sensitive (lysed) versus resistant (not lysed) cells present after treatment with active guinea pig serum relative to cells treated with heat-inactivated serum (HI). Data points represent means and SEM of results from 3 flasks collected and analyzed on a single day (LIT and TAU; *n* = 3) or from sets of 3 flasks collected on 2 different days (RPMI medium; *n* = 6). Spheromastigotes are rounded trypanosomes that appear with extended starvation but that revert to epimastigotes with the next feeding (64).

Our investigation required axenic insect culture conditions that would provide cells of multiple insect-stage morphologies. Epimastigotes were obtained from culture in liver infusion tryptose (LIT) medium (42) containing 10% fetal bovine serum (FBS). LIT medium allows the continuous division of epimastigotes. We found, as reported previously (43), that stationary *T. cruzi* strain CL Brener grown continuously in LIT medium does not contain appreciable numbers of metacyclic trypomastigotes, as assayed by cellular morphology (Fig. 1A and B; see Fig. S1 in the supplemental material).

10.1128/mSphere.00051-16.2 Figure S1 Morphology of *T. cruzi* during metacyclogenesis. (A) Schematic of *T. cruzi* metacyclogenesis. The nucleus is indicated in blue and the kinetoplast in orange. The posterior side of cells is at the top of the schematic. (B) Confocal microscopic images of *T. cruzi* CL Brener stages in day 8 RPMI cultures. The top panels show DAPI fluorescence, and the lower panels show transmitted light (T-PMT). Bar (shown in DAPI images), 2 mm. Download Figure S1, EPS file, 2.8 MB. Copyright © 2016 Shaw et al. 2016 Shaw et al. This content is distributed under the terms of the Creative Commons Attribution 4.0 International license.

In obtaining metacyclic trypomastigotes, the purity of the culture was secondary to the concern that cell-stage separation procedures such as chromatography do not themselves trigger changes in RNA levels (44). In our method, early exponential-growth cells from LIT medium are transferred to Roswell Park Memorial Institute medium (RPMI medium) with a final concentration of approximately 1% serum. Although initially provided with glucose and amino acids, cells incubated in RPMI medium even for extended times did not replicate (Fig. 1B). Rather, some of the cells underwent metacyclogenesis, and others exhibited characteristics of cell starvation (45–47). The percentage of differentiated cells in culture peaked 8 days after culture initiation (Fig. 1A and C). Differentiated cells include both metacyclic trypomastigotes and cells characterized as “intermediate” (48) (also visually defined in Fig. S1 in the supplemental material), with incomplete kinetoplast posterior migration. In our hands, incubation in RPMI medium stimulated CL Brener metacyclogenesis better than the existing method of a short period of abrupt starvation in a low-pH medium followed by return of glucose and certain amino acids (49, 50) (not shown). The gradual nutrient reduction is also presumably more biologically relevant. Enough differentiated cells are obtained to explore changes in gene expression without separating the metacyclic trypomastigotes from the undifferentiated cells in culture by chromatography (51).

We suspected an underreporting of metacyclic cells in the RPMI culture due to the relatively poor adherence of this stage onto microscope slides. Therefore, we estimated the portion of cells undergoing metacyclogenesis utilizing a biological method: the complement-mediated lysis of CL Brener epimastigotes (Fig. 1D). Approximately 40% of cells grown 8 days in RPMI culture escaped lysis. Furthermore, surviving cells exhibited movement and morphological characteristics of metacyclic trypomastigotes. Based on these two criteria, the cells were characterized as metacyclic trypomastigotes.

Since the day 8 RPMI culture still contained mixed stages, we employed a separation technique to acquire subcultures enriched for certain stages. The top fraction of an undisturbed day 8 RPMI culture contains primarily differentiated cells, while the bottom portion contains primarily epimastigotes (Fig. 1C and D). Thus, we have established a culture system to examine gene expression of populations enriched for either epimastigotes or intermediate/metacyclic trypomastigotes from a single culture exposed to the same nutrient stresses among other factors.

### Certain mitochondrial RNA abundances are altered in cells incubated in medium promoting differentiation.

**FIGURE 2:**
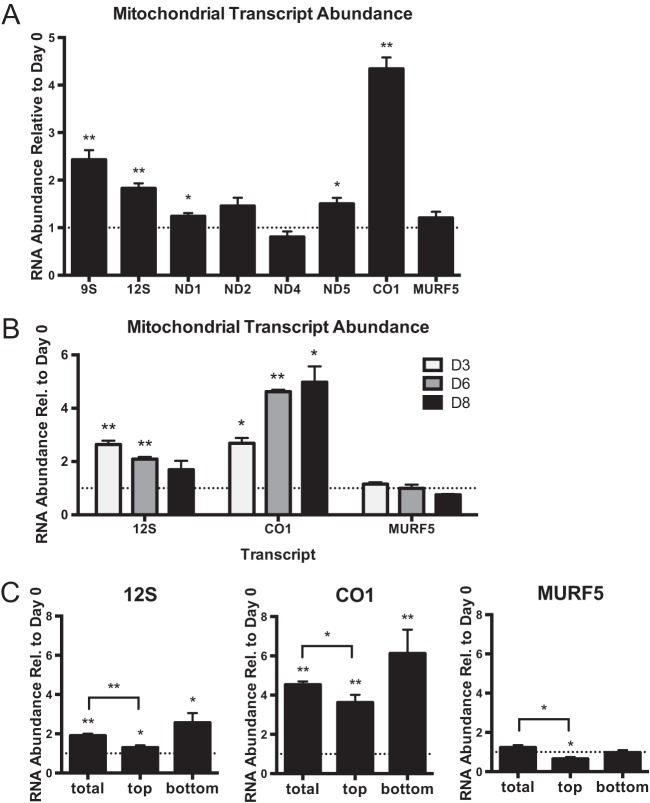
Mitochondrial RNA abundances of never-edited RNAs in insect stage differentiation culture. (A) Abundances of all eight never-edited mitochondrial transcripts were measured by qRT-PCR. Data points represent means and SEM of results from 8 to 9 flasks (total) collected in batches of replicate flasks on 4 different days. (B) Abundances of 12S, CO1, and MURF5 transcripts were measured by qRT-PCR over an 8-day time course. Undisturbed growth in RPMI medium at each time point was compared to day 0 levels in LIT medium. Data points represent means and SEM of results from 3 replicate flasks. (C) The same transcripts were measured by qRT-PCR in day 8 RPMI cultures (total) and the top and bottom fractions of a day 8 RPMI culture (top and bottom). Data represent means and SEM of results from 9 to 12 flasks (total) collected in batches of replicate flasks on 4 different days; these included RNA samples used as described for panel B for day 0 LIT and day 8 total data. For all, *y*-axis data represent the ratio of transcript abundance at the stated day of undisturbed growth in RPMI medium to the abundance in day 0 exponentially growing epimastigotes. Asterisks (**, *P* < 0.001; *, *P* < 0.05) indicate results of unpaired *t* test comparisons to day 0 epimastigotes, unless otherwise indicated. The dotted line on the graph represents a *y* value of 1 (no change relative to day 0). Normalization was to the mean abundance of *TERT* and *PFR2*.

We first examined whether expression of the two rRNAs and six mRNAs that are never edited was altered in cells after an 8-day incubation in RPMI medium. Development of next-generation sequencing of U insertion/deletion mitochondrial genomes is still ongoing (52, 53). As 20 maxicircle genes in total represent a very manageable number, we analyzed levels of individual RNAs by quantitative reverse transcription-PCR (qRT-PCR). We found significant differences in the abundances of some mRNAs and rRNAs in comparing levels in the 8-day RPMI culture to that of the exponentially growing epimastigote starting culture (day 0 LIT; Fig. 2A). In particular, 9S and 12S rRNA plus COI mRNA encoding a subunit of complex IV increased in abundance. Others, such as mRNAs encoding complex I components and maxicircle unidentified reading frame 5 (MURF5), differed very little if at all.

We next determined whether the observed changes in mRNA abundances between cells in differentiation culture and exponentially growing epimastigotes coincided with the appearance of metacyclic trypomastigotes. We selected a subset of RNAs to examine: 12S and COI, which exhibited increased abundance in the day 8 RPMI culture, and MURF5, which exhibited no such difference. Consistent with the detected differences in overall expression, each assayed transcript had its own temporal expression profile during incubation in RPMI medium (Fig. 2B). 12S levels increased by 3 days but then decreased over the remaining time points, CO1 levels continuously increased over time, and MURF5 mRNA levels were constant throughout the experiment. Therefore, a strict temporal relationship between 12S, COI, or MURF5 RNA abundances and the appearance of metacyclic trypomastigotes in culture does not exist.

We then asked whether RNA abundance changes in the RPMI culture occur primarily in differentiated cells (both intermediate and true trypomastigote morphologies) or in epimastigotes, utilizing our stage-enriched differentiation culture fractionation. Again, we compared the level in each 8-day RPMI culture fraction to that of the exponentially growing epimastigote starting culture (day 0 LIT). 12S and CO1 transcript expression was upregulated to the greatest extent in the day 8 RPMI medium epimastigote-enriched fraction (bottom) and to a lesser extent in the metacyclic-trypomastigote-enriched fraction (top), relative to the levels seen with the exponentially growing day 0 LIT epimastigotes (Fig. 2C). As the degree of change in relative abundance for 12S and CO1 was less in the metacyclic-trypomastigote-enriched fraction than in the total culture, the observed increases were unlikely to have been part of differentiation expression remodeling. Rather, the results suggest that these increases represent an epimastigote-specific and possibly transient response to environmental changes, such as the lower nutrient levels that are present in RPMI medium at later time points. There was, however, a slight but statistically significant decrease in relative MURF5 abundance in only the metacyclic-trypomastigote-enriched fraction, which could represent a life-stage-specific difference in expression.

As trypanosome mitochondrial transcription is thought to be largely constitutive (18), changes in levels of never-edited mRNAs and rRNAs can be attributed to changing transcript stability. In contrast, levels of mature, translatable edited mRNAs may be controlled by the speed or efficiency of editing in addition to their stability. We examined the relative RNA abundances of edited mRNAs in order to analyze more mitochondrial genes and also to seek evidence of editing regulation. We analyzed both pre-edited and edited forms of the mRNA, which we discerned by the use of specific qRT-PCR primers, as is common in *T. brucei* mRNA expression analysis studies (19, 20, 54–56). Three edited *T. cruzi* mRNA sequences are available with which to design primers: CO2, CO3, and A6. We determined the edited sequences of two others: CYb and MURF2.

**FIGURE 3:**
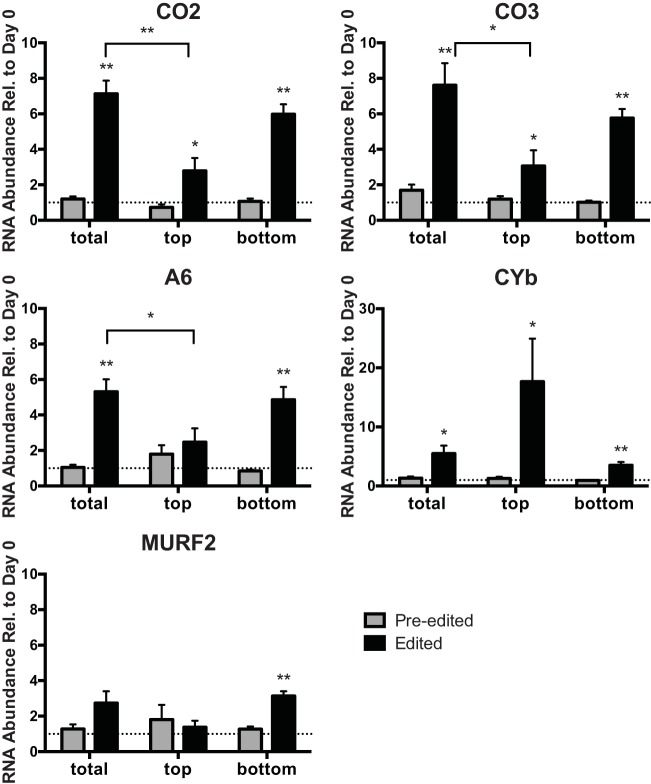
Regulation of editing in insect stage differentiation culture. Abundances of pre-edited (gray) and edited (black) forms of each RNA were measured by qRT-PCR in day 8 RPMI cultures (total) and the top and bottom fractions of a day 8 RPMI culture (top and bottom). The same RNA samples were utilized as described for Fig. 2C. *y* axis data represent ratios of transcript abundance after undisturbed growth in the indicated culture medium to the abundance in day 0 epimastigotes. The *y*-axis scale is different for CYb. Asterisks (**, *P* < 0.001; *, *P* < 0.05) indicate results of an unpaired *t* test in comparisons to day 0 epimastigotes, unless otherwise indicated. The dotted line on the graph represents a *y* value of 1 (no change relative to day 0). Normalization was to the mean abundance of *TERT* and *PFR2*.

We measured the relative abundances of these five mRNAs in total culture and in culture fractions enriched for epimastigotes and metacyclic trypomastigotes compared to exponentially growing epimastigotes (Fig. 3). There were no statistically significant increases in levels of pre-edited mRNAs; however, changes were evident in total culture for all five edited mRNAs. For four of these five mRNAs, namely, CO2, CO3 (encoding complex IV components), A6 (encoding a complex V component), and MURF2 (maxicircle unidentified reading frame 2), the magnitude of increase was less in the metacyclic-trypomastigote-enriched culture than in the total culture and the epimastigote-enriched culture. Therefore, as with 12S and CO1, edited CO2, CO3, A6, and MURF2 levels are increased in differentiation culture, with epimastigotes exhibiting the greatest increases. In contrast, the metacyclic-trypomastigote-enriched fraction demonstrated greater relative abundance increases in edited CYb than the total culture, and the epimastigote-enriched fraction showed a lower relative abundance increase in edited CYb. In summary, the abundances of the five analyzed edited mRNAs were increased in the differentiation culture, with the greatest increases in epimastigotes for CO2, CO3, A6, and MURF2 and the greatest increases in metacyclic trypomastigotes/intermediate cells for CYb.

### Nutrient starvation is a signal for increases in abundance of certain mRNAs and rRNAs.

**FIGURE 4:**
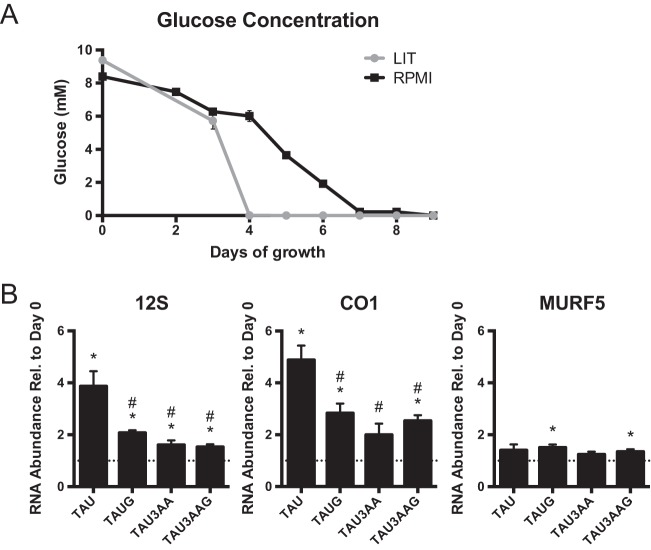
Mitochondrial RNA abundance changes in response to glucose and amino acid deprivation. (A) Glucose levels in undisturbed cultures of *T. cruzi* in LIT and RPMI medium over 9 days. Data points represent means and SEM of results from 3 replicate flasks (total) collected on 2 different days (*n* = 6). (B) 12S, CO1, and MURF5 mitochondrial transcript abundances were measured by qRT-PCR. *T. cruzi* was cultured for 1 day in TAU or in TAU with added glucose (TAUG) or in TAU plus 3 amino acids (TAU3AA) or in TAU with both glucose and 3 amino acids (TAU3AAG). Data points represent means and SEM of results from 3 replicate flasks. *y*-axis data represent ratios of transcript abundance after undisturbed growth in the indicated culture medium to the abundance in day 0 exponentially growing epimastigotes. Asterisks (**, *P* < 0.001; *, *P* < 0.05) indicate results of an unpaired *t* test in comparisons to day 0 epimastigotes. #, *P* < 0.05 (results of an unpaired *t* test in comparisons to TAU). The dotted line on the graph represents a *y* value of 1 (no change relative to day 0). Normalization for panel B data was to the mean abundance of *TERT* and *PFR2*.

We determined environmental changes in the differentiation culture responsible for initiating RNA abundance increases. Nutrient starvation plays a role in metacyclogenesis (47), and trypanosomes in insects experience fluctuations in nutrient availability (32). These fluctuations include transition from utilization of *T. cruzi*’s favored nutrient source, glucose, to utilization of amino acids, so we first determined the rate of depletion of glucose in the differentiation culture (Fig. 4A) to see if its disappearance correlated with mRNA abundance increases. Cells grown in RPMI medium are depleted of glucose by day 7. As this timing is inconsistent with the period when we detected increases in RNA abundances (Fig. 2B), glucose depletion alone is likely not the trigger for changes in RNA abundance.

We determined if we could observe RNA abundance changes in 12S, CO1, and MURF5 in culture depleted of both primary nutrient sources of insect-stage medium: glucose and amino acids. The medium triatomine artificial urine (TAU) is a buffered salt solution completely lacking nutrients (49); yet, remarkably, *T. cruzi* epimastigotes survive in this medium for extended time periods. Figure 4B shows that 24 h after exponentially growing epimastigotes were transferred to TAU, 12S and CO1 exhibited 4-fold abundance increases, and MURF5 showed a small, nonsignificant increase in abundance. By performing the same experiment except incubating in TAU that also contained glucose (TAUG) or amino acids (TAU3AA) or both (TAU3AAG), we tested whether the presence of either nutrient abrogates the abundance increase in mitochondrial rRNA or mRNA (Fig. 4B). In fact, adding one or the other or both nutrient sources significantly dampened the response of 12S and CO1 levels to TAU incubation. Therefore, epimastigotes incubated in TAU increase levels of mitochondrial RNAs mainly in response to total nutrient depletion. However, since mild abundance increases still occurred in the TAUG, TAU3AA, and TAU3AAG media, additional environmental factors also affect mitochondrial RNA abundances.

**FIGURE 5:**
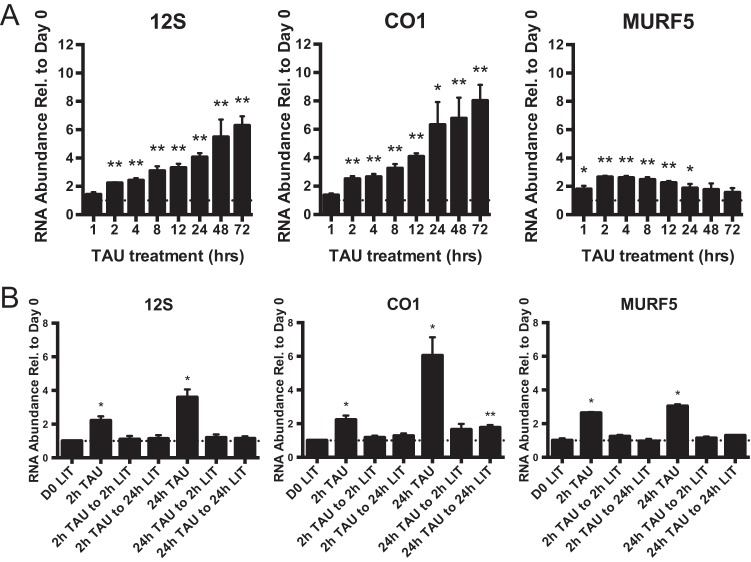
Mitochondrial RNA abundance upon starvation and nutrient return. (A) Transcript abundances were measured by qRT-PCR for TAU culture at various time points over 72 h of growth. For both panel A and panel B, data points represent means and SEM of results from 5 to 7 replicate flasks (total) collected on 3 different days. (B) Transcript abundances were measured by qRT-PCR in cultures starved for 2 or 24 h and returned to nutrient-rich LIT medium for 2 or 24 h. Data points represent means and SEM of results from 4 replicate flasks (total) collected on 2 different days. *y*-axis data represent ratios of transcript abundance after undisturbed growth in the indicated culture medium to the abundance in day 0 epimastigotes. Asterisks (**, *P* < 0.001; *, *P* < 0.05) indicate results of an unpaired *t* test in comparisons to day 0 epimastigotes, unless otherwise indicated. The dotted line on the graph represents a *y* value of 1 (no change relative to day 0). Normalization was to the mean abundance of *TERT* and *PFR2*.

The temporal nature of mitochondrial gene expression changes in epimastigotes under the TAU starvation condition (Fig. 5) was also analyzed. We already found significant increases in the abundances of 12S, CO1, and MURF5 transcripts 2 h after transfer to TAU. 12S and CO1 continued to consistently increase in expression over the 72-h incubation period of the experiment (Fig. 5A). In contrast, while MURF5 had already increased in abundance at 1 h following transfer to TAU, expression levels peaked at 2 to 4 h and relative abundance decreased in later time points. RNA abundance increases were reversible; regardless of whether cells were in TAU for 2 or 24 h, upon return to nutrient-rich LIT medium, levels of 12S and CO1 returned to normal within 2 h (Fig. 5B). Similar results were obtained when cells were transferred to TAU3AAG rather than LIT medium following TAU starvation (see Fig. S3 in the supplemental material).

10.1128/mSphere.00051-16.3 Figure S2 Mitochondrial RNA abundance changes during *T. cruzi* metacyclic differentiation. (A) Average nonnormalized abundances of 4 possible normalization RNAs (*TERT*, *actin*, *PFR2*, and 18S) were measured by qRT-PCR. The average nonnormalized abundances of day 0 LIT medium, day 8 RPMI medium (total), or the top and bottom fractions of day 8 RPMI cultures are shown. (B) Average nonnormalized abundances of day 0 LIT and h 1 to h 72 TAU cultures for the same 4 RNAs measured by qRT-PCR. *y*-axis data represent arbitrary units (AU) based on each standard curve, with the most concentrated point on the curve set to 1. Data points represent means and SEM of results from 9 to 12 replicate flasks collected on 4 different days. Asterisks (**, *P* < 0.001; *, *P* < 0.05) indicate results of an unpaired *t* test in comparisons to day 0 epimastigotes. Download Figure S2, EPS file, 0.9 MB. Copyright © 2016 Shaw et al. 2016 Shaw et al. This content is distributed under the terms of the Creative Commons Attribution 4.0 International license.

**FIGURE 6:**
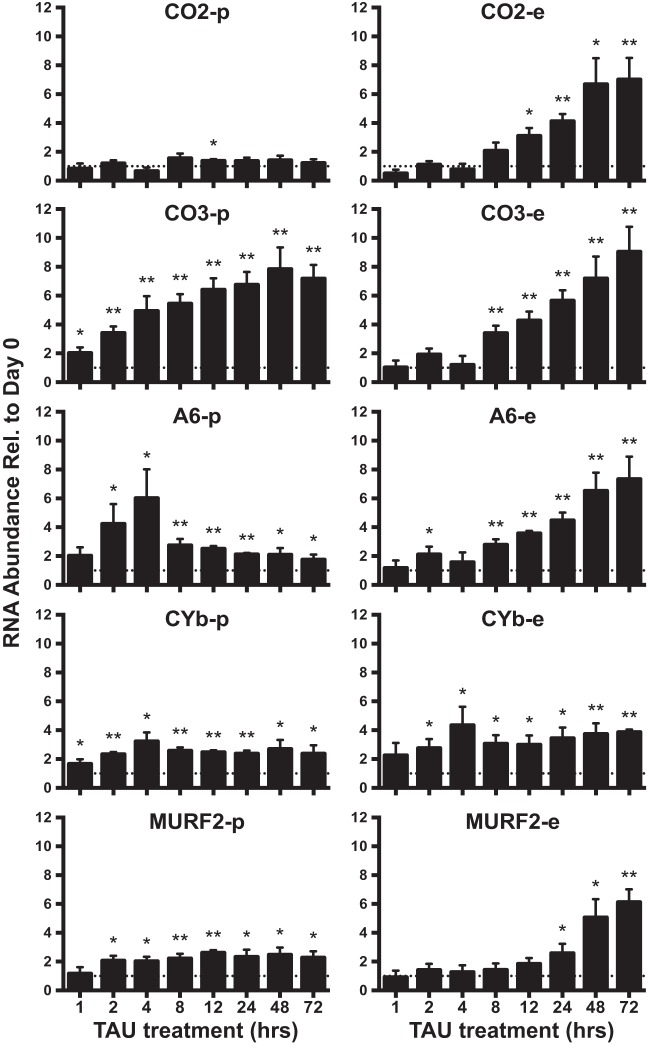
Starvation-dependent changes in edited mitochondrial RNA abundances. Abundances of pre-edited (-p) and edited (-e) forms of each RNA were measured by qRT-PCR in TAU culture at various time points over 72 h of growth. The RNA samples utilized were the same as those described in the Fig. 5 and 7 legends. *y*-axis data represent ratios of transcript abundance after undisturbed growth in the indicated culture medium to the abundance in day 0 epimastigotes. Asterisks (**, *P* < 0.001; *, *P* < 0.05) indicate results of an unpaired *t* test in comparisons to day 0 epimastigotes. The dotted line on the graph represents a *y* value of 1 (no change relative to day 0). Normalization was to the mean abundance of *TERT* and *PFR2*.

10.1128/mSphere.00051-16.4 Figure S3 Recovery of starvation response in TAU3AAG. Transcript abundances were measured by qRT-PCR from cultures starved for 2 or 24 h (TAU), after which TAU was replaced with TAU3AAG for 2 or 24 h. Data points represent means and SEM of results from 2 replicate flasks. Data points for day 0 LIT medium and some of the data points for h 2 and h 24 TAU are also included in Fig. 6B. *y*-axis data represent ratios of transcript abundance after growth under the indicated conditions to the abundance in day 0 epimastigotes. Asterisks (**, *P* < 0.001; *, *P* < 0.05) indicate results of an unpaired *t* test in comparisons to day 0 epimastigotes, unless otherwise indicated. The dotted line on the graph represents a *y* value of 1 (no change relative to day 0). Normalization was to the mean abundance of TERT and PFR2. Download Figure S3, EPS file, 0.8 MB. Copyright © 2016 Shaw et al. 2016 Shaw et al. This content is distributed under the terms of the Creative Commons Attribution 4.0 International license.

We extended our study of nutrient starvation of epimastigotes in TAU to the examination of edited mRNA abundances. Three of the five mRNAs examined, edited CO2, CO3, and A6, exhibited similar patterns of increasing abundance over time in TAU, and the changes mirrored the results seen with never-edited CO1 (Fig. 6). Edited MURF2 also displayed a continual increase in abundance over time and yet responded more gradually than A6, CO2, and CO3. However, a different pattern was observed for edited CYb. After an initial increase, levels of CYb remained at a consistently higher level over the time course. These results support our earlier assertion that abundance changes in the RPMI differentiation medium represent a response to nutrient depletion by epimastigotes.

### Nutrient deprivation does not increase nucleus-encoded ETC component mRNA abundances.

**FIGURE 7:**
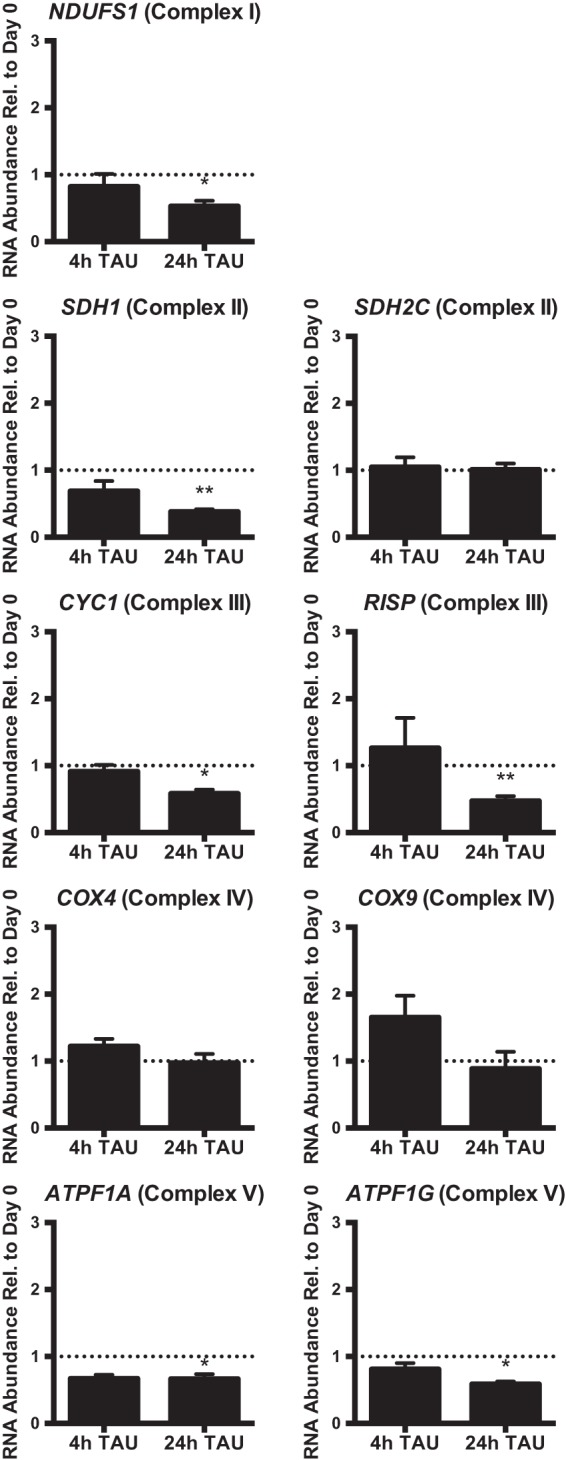
Abundance of nucleus-encoded transcripts encoding ETC complex components. Nucleus-encoded *NDUFS1* (encoding complex I ferredoxin), *SDH2C* and *SDH1* (complex II succinate dehydrogenase iron-sulfur subunit and succinate dehydrogenase flavoprotein), *CYC1* and *RISP* (complex III cytochrome *c*_1_ and Rieske iron-sulfur protein), *COX4* and *COX9* (complex IV cytochrome *c* oxidase subunits IV and IX), and *ATPF1A* and *ATPF1G* (ATP synthase F1 alpha subunit and gamma subunit) transcript abundances were measured by qRT-PCR in TAU culture at 4 and 24 h (h) of growth. The RNA samples utilized were the same as those described in the Fig. 5 and 6 legends. *y*-axis data represent ratios of transcript abundance after undisturbed growth in the indicated culture medium to the abundance in day 0 epimastigotes. Asterisks (**, *P* < 0.001; *, *P* < 0.05) indicate results of an unpaired *t* test in comparisons to day 0 epimastigotes. The dotted line on the graph represents a *y* value of 1 (no change relative to day 0). Normalization was to the mean abundance of *TERT* and *PFR2*.

ETC complexes I, III, IV, and V include both mitochondrion- and nucleus-encoded subunits (33). We determined if nutrient depletion also results in changes of mRNAs from nuclear genes encoding ETC components. We selected mRNAs from nine nucleus-encoded ETC subunits representing all five complexes (33). In contrast to most mitochondrial mRNAs, nucleus-derived mRNAs did not increase in abundance at 4 or 24 h in TAU. In fact, six of these RNAs demonstrated significant 2-fold depletions by 24 h (Fig. 7). We conclude that abundance increases of mitochondrion-encoded ETC component subunits such as CO1 during starvation are not matched by corresponding abundance increases in nucleus-encoded subunit mRNAs. If increases in nucleus-encoded subunit abundances do occur, they must be a result of changes at the level of translation and/or protein stability control.

### Nutrient deprivation results in small or negligible changes to select nucleus-encoded ETC component protein abundances.

The technical inability to generate antibodies to maxicircle-encoded trypanosome proteins prevents us from directly measuring whether their abundance parallels their mRNA levels. Fortunately, we are able to detect certain nucleus-encoded ETC subunits with specific antibodies. We hypothesized that mitochondrial mRNA levels may be the limiting factor in ETC complex generation. In this case, greater mitochondrial mRNA abundances in starved epimastigotes may result in greater abundances of ETC complexes even if levels of nucleus-encoded subunit mRNAs do not increase. A greater availability of binding partners for nascent nucleus-encoded proteins can stabilize them.

**FIGURE 8:**
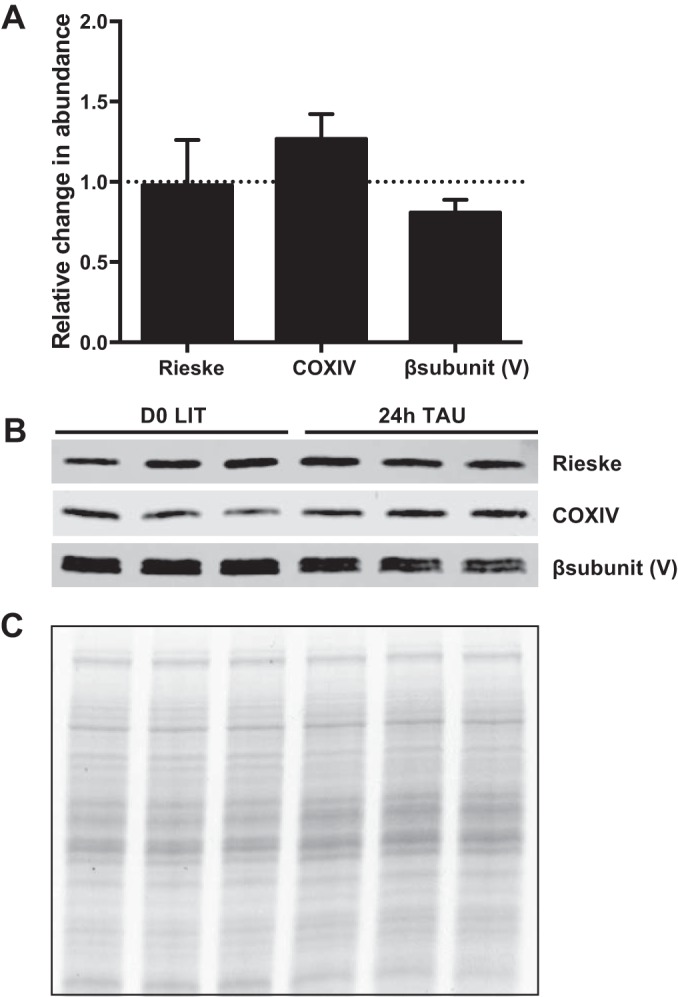
Abundance of nucleus-encoded ETC complex components. *T. cruzi* crude mitochondrial protein preparations from cells grown in LIT medium or 24 h in TAU were probed with nucleus-encoded *T. brucei* Rieske (complex III) antibody, *Leishmania major* COXIV (complex IV) antibody, or *T. brucei* beta subunit (complex V) antibody. Each of three biological replicates was loaded in triplicate for immunoblot analysis. Loading controls used for normalization were Coomassie-stained gels of identical triplicate loads. (A) Quantitation of normalized immunoblot signals. (B) Representative immunoblots of 20 µg of mitochondrial protein per lane loaded in triplicate. (C) Coomassie stain of protein gel of the same mitochondrial protein preparation, loaded in triplicate, used for normalization.

In order to preliminarily test this hypothesis, we first determined that antibodies recognizing *T. brucei* ETC subunits cross-reacted with *T. cruzi* homologues (see Fig. S4 in the supplemental material). We then tested levels of one subunit each from complexes III, IV, and V in a mitochondrial extract from exponentially growing epimastigotes (LIT) or after transfer to TAU for 24 h (Fig. 8). Upon normalization, we could not detect any increase in expression of the complex III or V subunits of the nucleus-encoded proteins; in fact, levels of the complex V subunit possibly decreased. We did consistently observe a modest (20% to 30%) but statistically nonsignificant increase in expression after TAU incubation of nucleus-encoded COXIV of complex IV despite its unchanged mRNA levels at 24 h of growth in TAU (Fig. 7). This result does not rule out the possibility that mitochondrion-encoded subunits may be limiting in at least some instances. However, the changes in overall ETC complex abundances may be too subtle to accurately capture with the methods employed here. Considering the lack of abundance increase of the complex III and V nucleus-encoded subunits tested, alternative biological outcomes for increases in levels of certain mRNAs under conditions of nutrient starvation, other than overall changes in ETC complex abundances, are also possible.

10.1128/mSphere.00051-16.5 Figure S4 Cross-reactivity of ETC subunit antibodies to *T. cruzi* homologues in mitochondrial extracts. A total of 150 (Rieske or COXIV) or 50 (β subunit) μg of mitochondrial extract from *T. brucei* or *T. cruzi* was loaded on SDS PAGE gel, run, and transferred to nitrocellulose membrane that was probed with indicated antibody. Download Figure S4, TIF file, 0.8 MB. Copyright © 2016 Shaw et al. 2016 Shaw et al. This content is distributed under the terms of the Creative Commons Attribution 4.0 International license.

## DISCUSSION

This work is an initial foray into revealing *T. cruzi* mitochondrial gene expression and its regulation. We focused on regulation during development and nutritional stress in the insect developmental stages. We demonstrate for the first time that expression of the *T. cruzi* mitochondrial genome at the RNA level has the potential to respond to developmental and/or environmental cues. When cells from metacyclic-trypomastigote-enriched fractions exhibit higher increases in RNA abundances than either the total culture or epimastigote-enriched fractions in our RPMI medium differentiation culture, such as with CYb, it suggests a life-stage-specific change. If we had instead seen that abundances of mitochondrial RNAs increased to similar degrees in the two fractions and in the unfractionated culture, it would have suggested that increased mitochondrial RNA abundances represent a stress response that is cell stage independent. With the exception of CYb, the response of mRNAs was different from that associated with either of these two scenarios. mRNA abundances were mainly correlated with epimastigotes transitioning from the fed state to the starved state, which is indicative of a starvation stress response within this life stage. This demonstration of malleability of epimastigote mitochondrial gene expression raises the issue of whether any mammalian life stages, presumably also experiencing differing levels of nutrient availability, experience changes in mitochondrial gene expression.

The expression phenotype observed in RPMI differentiation medium and under starvation conditions is a real targeted response. We have carefully considered normalization (see Fig. S2 in the supplemental material), so we know that it is the mitochondrial mRNAs and not normalization genes that change in abundance. The consistent levels of the majority of the nine analyzed nucleus-encoded ETC subunit mRNAs (Fig. 7) also suggest that the increased abundance of our targets is not simply an experimental artifact. Additionally, not all mitochondrial gene products exhibit changes in gene expression when starved. Therefore, the expression pattern does not represent a global nonspecific response, and likely there is a reason certain mRNAs are more abundant. Finally, for a subset of related edited transcripts, abundance changes followed the same temporal patterns despite their pre-edited transcript forms showing differing temporal expression patterns (Fig. 6). A potential explanation is that the same protein complex(es) is driving rates of editing of these particular mRNAs.

The most likely mechanism for starvation-induced abundance increases occurring more strongly in the edited than the pre-edited RNA populations is control exerted at the level of speed or efficiency of RNA editing. Of course, differential stability is also a possibility, as this appears to be the mode of increasing CO1, 9S, and 12S abundance. Recent work in *T. brucei* suggests that translation is a key control point of mitochondrial gene expression (57). This may or may not be true in *T. cruzi*, but our study only touched on this regulatory level. Experiments involving ribosome purifications and analysis of the extension of 3′ nonencoded tails (ex-tailing) that is associated with translatable mRNAs (58) are certainly warranted and can utilize the experimental system that we have developed here. If future studies demonstrate a major role for translational control in *T. cruzi* gene expression, they will not negate that changes in control of stability and editing also occur and may contribute to overall protein product expression patterns.

A major issue emerging from this study is the impact of changes in mitochondrial gene expression on *T. cruzi* biological function. The starvation-induced changes that we observed for CO1, CO2, and CO3 mRNA abundances in *T. cruzi* are of lower magnitude than the life-stage abundance differences between these mRNAs in *T. brucei*, where complex IV is absent in the bloodstream stage, which relies entirely on glycolysis for energy metabolism (29, 33). The biological consequences of *T. cruzi* mitochondrial gene expression changes could therefore be relatively subtle.

One scenario is that the translational machinery of the *T. cruzi* mitochondrion could be inefficient during starvation and require more input to maintain adequate protein production. A remodeling of the ETC in which changes in individual complex abundance at the protein level occur but are less than 30% in magnitude and are hard to quantitatively capture also makes sense, since constituent complexes likely function in all *T. cruzi* life stages (5, 33–36). Even small changes in ETC complex abundances could matter for survival of a population under starvation conditions. Conversely, ETC complexes may actually experience a change in mitochondrion-encoded versus nucleus-encoded subunit ratios. Interestingly, at least one ETC protein is not physically associated with complex V in *T. brucei* as it is in other model systems (59). This demonstrates that increases in mitochondrial but not nuclear ETC proteins that could lead to ETC functional changes are possible without compromising the physical integrity of complex I to complex V.

Another possibility is that the regulatory pathway resulting in altered mitochondrial gene expression in response to starvation is present and functional both when it is needed and when it is not. There may be other life stages or situations involving starvation of *T. cruzi*, perhaps in the context of its native environment, where ETC complex nuclear subunits do increase in abundance to meet the increased expression of the mitochondrion. Finally, trypanosome mitochondrial gene products may have evolved functions outside their established role as ETC complex subunits in these highly diverged eukaryotes and their increased expression is for the non-ETC-related roles. In this final scenario, an increase in the abundance of nuclear ETC complex subunits would not be expected. If, indeed, ETC expression or modification results from changes to mitochondrial gene expression, it may be necessary for an oxidative stress response as well as for metabolic changes. Intriguingly, complex III activity in *T. cruzi* bloodstream trypomastigotes is higher than that of epimastigotes, and yet complex IV activity is not increased (36) and thus has been hypothesized to be an adaptive oxidative stress response. In our study, CYb, encoding a complex III subunit, was the only mRNA whose abundance increased mainly in the metacyclic-trypomastigote-enriched culture fraction, suggesting the possibility of higher levels of complex III in metacyclic as well as bloodstream trypomastigotes.

Finally, regardless of the biological relevance of the mRNA abundance increases characterized here, this experimental system is a new model in which to probe editing and stability regulation. As new technologies are increasing the genetic malleability of *T. cruzi* (60), mechanistic studies that were previously limited to *T. brucei* are now possible. For example, in our *T. cruzi* starvation model systems, we can ask what additional proteins are associated with editing complexes upon epimastigote starvation or whether the changes to stability of CO1 upon starvation depend on polyadenylation or uridylation activity. In summary, investigations into *T. cruzi* mitochondrial gene expression are long overdue and can lead to a better understanding of both *T. cruzi* biology and the regulation of trypanosome gene expression in general.

## MATERIALS AND METHODS

### Cell culture.

The *T. cruzi* CL Brener strain (61) was from Roberto Docampo (University of Georgia). Cells were maintained in LIT medium (42) containing 10% fetal bovine serum (FBS) in humidified CO_2_ incubators at 27°C. Exponential-phase cells were routinely diluted 1:10 in fresh LIT medium every 2 to 3 days. For metacyclogenesis, 1 ml of exponential-phase cells in LIT medium was added to 10 ml of RPMI medium without FBS and left undisturbed for 8 days. To purify RPMI culture fractions, the flask was carefully tilted upright without disturbing the consolidated bottom material, and the top 7 ml was withdrawn as the top layer. The bottom 4 ml and attached cells were collected as the bottom layer. For nutritional starvation, exponential-phase cells were collected and resuspended in TAU. TAU was supplemented with 10 mM l-proline, 50 mM l-glutamate, and 2 mM l-aspartate to generate TAU plus 3 amino acids (TAU3AA), with 10-mM glucose to generate TAU plus glucose (TAUG), or with all 4 supplements to generate TAU3AAG (62). For growth assays, cells were mixed to dislodge attached cells and suspended cells were counted on a hemocytometer every 3 days in triplicate cultures.

### Microscopy.

Cells were collected from flasks, with aggressive mixing performed to dislodge attached cells when necessary, and resuspended at 2.5 × 10^7^ cells/ml in PBS-G (phosphate-buffered saline [PBS] plus 0.1% glucose). Cells were fixed with the addition of a 0.5 vol of 8% paraformaldehyde–PBS and incubated for at least 30 min at 4°C. Silicone isolators were used to form wells on slides to contain trypanosomes during processing (Grace Bio-Labs, Bend, OR). Cells in fixative were applied to poly-l-lysine-coated slides (30 min), permeabilized with PBS–0.5% NP-40 detergent (Igepal; 30 min), and washed with PBS-G. Cells were stained with 1 µg/ml 4′,6-diamidino-2-phenylindole (DAPI) for 30 min and washed with PBS-G. Slides were coverslipped with Vectashield Hardset (Vector Laboratories, Burlingame, CA) and left overnight. Images were captured on a Carl Zeiss LSM 710 microscope using a 63× oil differential interference contrast (DIC) objective. Images were processed using Carl Zeiss ZEN 2012 SP1 (black edition) 64-bit version 8.1 software. Epimastigotes were identified as cells showing a bar-like kinetoplast located anterior to the nucleus. Spheromastigotes were identified as circular cells without anterior-posterior polarity, with a short, encircling flagellum (63, 64). Intermediate cells were identified as cells in which the disk-shaped kinetoplast had become round and migrated at least as far as the center of the nucleus on its way to the posterior end of the cell, and metacyclic cells were identified as cells showing a circular kinetoplast located fully posterior to the nucleus in elongated cells (48). Both intermediate and metacyclic cells displayed an elongated morphology and ruffled flagella. Cells were counted manually using the cell-counting plugin in NIH ImageJ64 1.47v. At least 100 cells were counted for each sample in three or more cultures.

### Complement-mediated lysis.

Parasites (500,000) were collected by centrifugation and resuspended in 160 µl RPMI medium supplemented with 7 µM CaCl and 10 µM MgCl. A 40-μl volume of guinea pig serum or of heat-inactivated guinea pig serum was added, and the mixture was incubated at 37°C for 45 min. Ice-cold RPMI medium (800 μl) was added, and the tubes were stored on ice. Live cells were quantified in a Neubauer chamber. The fraction of cells remaining was calculated as the number of serum-treated cells remaining divided by the number of heat-inactivated serum-treated cells remaining. In all cases, triplicate flasks were analyzed, with an additional biological single replicate for all samples from RPMI medium-derived cultures.

### qRT-PCR.

RNA was isolated from cell pellets or attached cells using TRIzol reagent (Life Technologies, Grand Island, NY). RNA was digested with DNase using a DNAfree kit (Life Technologies), after which its integrity was verified by agarose electrophoresis. Total RNA (4 µg) was reverse transcribed to generate cDNA using a TaqMan reverse transcription kit (Life Technologies). Relative RNA abundances were determined by qRT-PCR using LightCycler 480 instrumentation and software version 1.5.1.62 (Roche, Indianapolis, IN). Samples were quantitated using the second-derivative-maximum method and fitted to a standard curve of five 4-fold serial dilutions of cDNA. Samples from a minimum of 4 separate cultures from at least two biological replicates (collected on different days) in duplicate wells (8 wells in total) were tested. Primer sequences are listed in Table S1 in the supplemental material along with each gene’s systematic identification number, with edited portions of CYb and MURF2 sequenced and deposited in GenBank, in order to design their primers. Statistical differences were compared using an unpaired *t* test, and significance was determined using the Holm-Sidak method, with alpha = 0.05. Each row was analyzed individually, without assuming a consistent standard deviation (SD).

10.1128/mSphere.00051-16.1 Table S1 qRT-PCR primer sequences. Download Table S1, DOCX file, 0.1 MB. Copyright © 2016 Shaw et al. 2016 Shaw et al. This content is distributed under the terms of the Creative Commons Attribution 4.0 International license.

Normalization of individual samples for real-time qRT-PCR analysis was analyzed. Because we did not know how rRNA levels typically used for normalization would respond to our conditions, we elected to analyze abundance trends of four potential normalization RNAs (three mRNAs and one rRNA) in nonnormalized samples (see Fig. S2 in the supplemental material). As only *PFR2* and *TERT* (65) normalization RNA abundances were not significantly different in RPMI medium (or TAU) compared in day 0 LIT medium, these two mRNAs were selected for normalization in LIT, TAU, and RPMI cultures.

### Glucose assay.

Culture medium was collected from cells at various time points and centrifuged. The supernatant was analyzed using an Amplex Red Glucose/glucose oxidase assay kit (Life Technologies). Fluorescence was read 20 min after color reagent addition on a SpectraMax M3 plate reader (Molecular Devices, Sunnyvale, CA) at 544-nm excitation and 590-nm emission. The quantity of glucose in medium was derived from a 7-point linear regression curve from 200 to 0.27 µM glucose. Culture medium was diluted to obtain values from within the standard range.

### Protein analysis.

Mitochondria were collected from approximately 5 × 10^8^ total cells using established methods (66). Protein was quantitated with a Pierce Micro BCA protein assay kit according to manufacturer’s instructions. Immunoblots were probed with *T. brucei* Rieske (67) (Tb09.211.4700; complex III) antibody (1:1,000 dilution), *Leishmania major* COXIV (complex IV; serum produced by Emanuela Handman, Walter and Eliza Hall Institute of Medical Research emeritus) antibody (1:1,500 dilution), or *T. brucei* beta subunit (67) (Tb927.3.1380; complex V) antibody (1:10,000 dilution). Detection of infrared signal from anti-rabbit secondary antibodies was performed using a Li-COR Odyssey Fc imaging system with the corresponding Image Studio software for analysis. The total protein extract of *T. cruzi* was also immunoblotted, but only the complex V antibody produced an appropriate signal, and results mirrored those seen with probing of mitochondrial proteins (not shown).

### Nucleotide sequence accession numbers.

Edited portions of CYb and MURF2 were sequenced and the sequences deposited in GenBank under accession no. KT312841 and KT312842, respectively.
