# Supplementary figures and images for: Mitochondrial Gene Expression Is Responsive to Starvation Stress and Developmental Transition in Trypanosoma cruzi
Source: mSphere. 2016 Apr 13;1(2):e00051-16. doi: 10.1128/mSphere.00051-16 (PMC4894683; doi:10.1128/mSphere.00051-16)

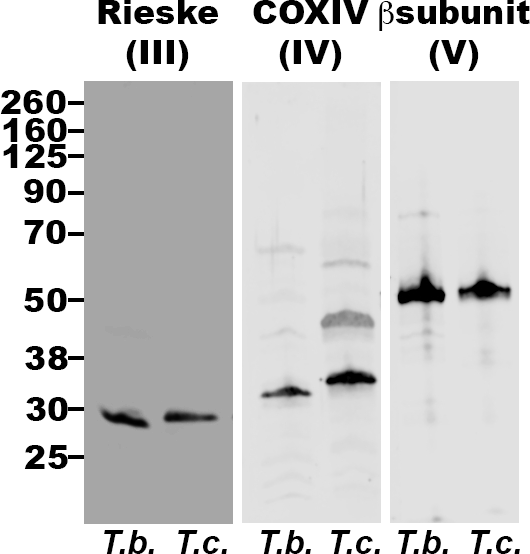

Supplement: Figure S4 [file sph002162067sf5.tif]
