# Supplementary material for: Mitochondrial Gene Expression Is Responsive to Starvation Stress and Developmental Transition in Trypanosoma cruzi
Source: mSphere. 2016 Apr 13;1(2):e00051-16. doi: 10.1128/mSphere.00051-16 (PMC4894683; doi:10.1128/mSphere.00051-16)
Supplement: Table S1 [file sph002162067st1.docx]

| **Name** | **Forward Primer** | **Reverse Primer** | **TriTrypDB or GenBank** |
| --- | --- | --- | --- |
| ***TERT*** | CGCTCCTCCGTTGTTGTACT | CAAACGGATGGTGTTGCTGG | TcCLB.509745.60 |
| ***Actin A*** | CAGAAGCCCCTATGAACCCC | ACTGCCTGAATGCCAACGTA | TcCLB.510127.79 |
| ***PFR2*** | GCTTATTGTCGCCGGGAAAA | CTCGCTCTCACACTCCTCAA | TcCLB.509319.109 |
| ***18S*** | CGGAATGGCACCACAAGAC | TGGTAAAGTTCCCCGTGTTGA | Tb927.2.1452 |
| **9s** | tgctatttaatgggtgtggaaat | tggcatccatttctgactaatat | DQ343645 |
| **12S** | ggcaagtcctactctcctttac | ttaatgcttgttaacctgctcg | DQ343645 |
| **ND1** | CAGGATTTGTTAGCCTTTGTGAA | AGCCGGCCCAACTCTAAG | DQ343645 |
| **ND2** | ACTCCTTCACTTTTGCTTCTTCC | CCCCATCCATTCTTCGTCAA | DQ343645 |
| **ND4** | cgtgtgactaccggaaatgc | tgctaatacaacactcatttcgg | DQ343645 |
| **ND5** | tctgtttggtttgatatcagga | acttataaaatgcctgcccaaca | DQ343645 |
| **CO1** | AGCCTAGTTACGTCTGCACT | TAACACCACCAGCCAAGACT | DQ343645 |
| **MURF5** | AAATGGGTTAATGATTTGGTT | CATTAATGTGTATGGTATTTAAC | DQ343645 |
| **A6-p** | CGTTTTTGCCAAGCTTAGAAGAAA | CTAAAAATTTCTCCCTTTTCAAAAAGCC | DQ343645 |
| **A6-e** | GATTTATTTTGGTTGCGTTTATTATG | CACAAACCAATAAACGAATATAAATCAAA | PMID: 8548658 |
| **CO2-p** | ATTGCAGTGTAATCATGTACACACATT | TTCATTACACCTACCCGGTTCTCT | DQ343645 |
| **CO2-e** | ATTGCAGTGTAATCATGTACACACATT | ATTTCATTACACCTACCCGGTATACAA | PMID: 8288582 |
| **CO3-p** | GAAACCAAGTGGGTTTAAACAAAG | TTTTCCCTCTCGAAACTCTCTCT | DQ343645 |
| **CO3-e** | TTGTTGTTTATTACGTGTTGTCCAATATTG | GTAAACAAAAAACAAAAGCAAACTCACAAC | EF058194 |
| **CYb-p** | AAATAAAAAGCGGAGAAAAGAGGAA | TCCATATATTCTATATAAGCAACCTGACA | DQ343645 |
| **CYb-e** | ATGTCGTGTATGTTATTTTTATTATTTTT | TCCATATATTCTATATAAGCAACCTGACA | KT312841 |
| **MURF2-p** | GGGATTAAGAAGTTTTGATTGAG | CAATATATAATCTAAATCAAATCATCAC | DQ343645 |
| **MURF2-e** | TAATGTTTGGTTGTTTTAATTTAGTTTTATTT | CAATATATAATCTAAATCAAATCATCAC | KT312842 |
| ***NDUFS1*** | TGTGGTATGCCGCGCCTCCGCT | GGCCCGCAGCGACACCCTCCA | TcCLB.509809.10 |
| ***SDH2C*** | ATTGAGCCTCTTGACCGTGA | CTTCGGGCAAGTGATACTGC | TcCLB.504949.30 |
| ***SDH1*** | TGGCAAATCATGTGCGAACA | GATTGCGTCCTTCCTTCGTC | TcCLB.511909.40 |
| ***CYC1*** | GCCGCATGACTTTCACTCAT | GGAAGCGTGTCTGTTGGTTT | TcCLB.511391.160 |
| ***RISP*** | GTGGTCAGACGAGTTCCTGA | CGCGTACTTTCCGTGCTTAT | TcCLB.510759.120 |
| ***COX4*** | CCCGCCGACCGCGCGGAGAT | GCGCCGCGCCGACGGGTCAT | TcCLB.506529.360 |
| ***COX9*** | TGCTGGCGGCGGGCCAAGTCA | GGCGCCCGGGGCTGGTCTGA | TcCLB.507611.280 |
| ***ATPF1A*** | CTCAGAACGTGGCGATGAAG | TGATTGAACAGCGCAACGAA | TcCLB.510609.40 |
| ***ATPF1G*** | AGGAAGCGGACCGTTATCAA | TTCTCAGTACTAGCGGCGTC | TcCLB.504069.80 |
